# Adult and Embryonic GAD Transcripts Are Spatiotemporally Regulated during Postnatal Development in the Rat Brain

**DOI:** 10.1371/journal.pone.0004371

**Published:** 2009-02-03

**Authors:** Anke Popp, Anja Urbach, Otto W. Witte, Christiane Frahm

**Affiliations:** Department of Neurology, Friedrich-Schiller-University, Jena, Germany; University of Washington, United States of America

## Abstract

**Background:**

GABA (gamma-aminobutyric acid), the main inhibitory neurotransmitter in the brain, is synthesized by glutamic acid decarboxylase (GAD). GAD exists in two adult isoforms, GAD65 and GAD67. During embryonic brain development at least two additional transcripts exist, I-80 and I-86, which are distinguished by insertions of 80 or 86 bp into GAD67 mRNA, respectively. Though it was described that embryonic GAD67 transcripts are not detectable during adulthood there are evidences suggesting re-expression under certain pathological conditions in the adult brain. In the present study we systematically analyzed for the first time the spatiotemporal distribution of different GADs with emphasis on embryonic GAD67 mRNAs in the postnatal brain using highly sensitive methods.

**Methodology/Principal Findings:**

QPCR was used to precisely investigate the postnatal expression level of GAD related mRNAs in cortex, hippocampus, cerebellum, and olfactory bulb of rats from P1 throughout adulthood. Within the first three postnatal weeks the expression of both GAD65 and GAD67 mRNAs reached adult levels in hippocampus, cortex, and cerebellum. The olfactory bulb showed by far the highest expression of GAD65 as well as GAD67 transcripts. Embryonic GAD67 splice variants were still detectable at birth. They continuously declined to barely detectable levels during postnatal development in all investigated regions with exception of a comparatively high expression in the olfactory bulb. Radioactive *in situ* hybridizations confirmed the occurrence of embryonic GAD67 transcripts in the olfactory bulb and furthermore detected their localization mainly in the subventricular zone and the rostral migratory stream.

**Conclusions/Significance:**

Embryonic GAD67 transcripts can hardly be detected in the adult brain, except for specific regions associated with neurogenesis and high synaptic plasticity. Therefore a functional role in processes like proliferation, migration or synaptogenesis is suggested.

## Introduction

GABA (gamma-aminobutyric acid), the main inhibitory neurotransmitter in the brain, is synthesized by glutamic acid decarboxylase (GAD). GAD exists in two isoforms termed GAD65 and GAD67 due to their molecular weights of 65 and 67 kDa, respectively. These enzymes are the products of two independently regulated genes sharing 65% sequence homology in rats [1]–[3]. Most GABAergic interneurons express both subtypes of GAD [4], [5] which are simultaneously detectable in the rat brain as early as embryonic day 17 [6]. GAD67 is found in axonal regions as well as in neuronal cell bodies, whereas GAD65 is mainly associated with synaptic terminals [7]. Therefore it has been suggested that GAD67 mostly provides a pool of GABA for general metabolic activity while GABA synthesized by GAD65 is likely to be more involved in synaptic transmission [8]. Mice lacking GAD65 are vital and do not exhibit changes in their brain GABA content though they have an increased susceptibility to seizures [9], [10]. In contrast, GAD67 knockout mice are not viable due to a cleft palate with following respiratory impairments and a dramatically reduced GABA level. However, their brains do not exhibit any recognizable structural defects [11].

During embryogenesis the mRNA coding for GAD67 is regulated by alternative splicing [12], [13]. At least two additional transcripts exist, I-80 and I-86 (summarized as EGAD), distinguished by insertions of 80 or 86 bp in GAD67 mRNA, respectively. The two inserts are identical with exception of the 6 bp at the 3′-end of the larger fragment containing an overlapping stop-start codon. The complete coding region of embryonic GAD messages comprises 1,860 (80 bp insert) and 1,866 bp (86 bp insert). Both embryonic transcripts code for a short enzymatically inactive GAD protein of 25 kDa (GAD25) which corresponds to the amino-terminal regulatory region of GAD67 and therefore has putative regulatory functions. Termination-reinitiation at the stop-start codon of I-80 additionally produces an enzymatically active protein of 44 kDa (GAD44) corresponding to the carboxy-terminal catalytic domain of GAD67 that contains the pyridoxal phosphate cofactor binding site [13].

GABA synthesized by GAD primarily acts as an inhibitory neurotransmitter but also serves trophic and metabolic functions [8], [14]–[17]. It is mainly synthesized by the adult GAD enzymes. But GAD44 was also shown to produce GABA [13]. Throughout brain development embryonic and adult GAD forms are expressed in a highly specific temporal manner [13], [18]. Embryonic GAD67 transcripts reach their maximum during embryonic development and decline to barely detectable levels shortly after birth [13], [19]. Conversely, GAD65 and GAD67 exhibit an increase within the first postnatal weeks [20], [21]. The existence of multiple forms of GAD, their localization in different cellular compartments [7], [22], and their confinement to certain developmental stages imply that they serve different functions.

In this context it is noteworthy that a strong re-expression of embryonic transcripts was found in adult brain under pathophysiological conditions. Szabo et al. revealed their expression in hippocampal granule cells after kainic acid administration [23] whereas we detected embryonic transcripts following stroke (A. Popp, unpublished data). This imitation of an embryonic expression pattern might be important for structural and functional reorganization following brain injury.

To date, nothing is known about the physiological relevance of the embryonic splice variants. In the embryonic rat brain they are mainly expressed in regions associated with proliferative but also migratory and post-mitotic cells [17]. To determine their expression level and localization in the postnatal rat brain and thus their potential role during postnatal development we, as a first step, investigated the postnatal expression (P1 to P90) of GAD-related mRNAs in cortex, hippocampus, cerebellum, and olfactory bulb (OB) of rats by means of qPCR and their spatiotemporal expression using radioactive *in situ* hybridization.

## Results

### Postnatal GAD65 and GAD67 mRNA expression

**Figure 1:**
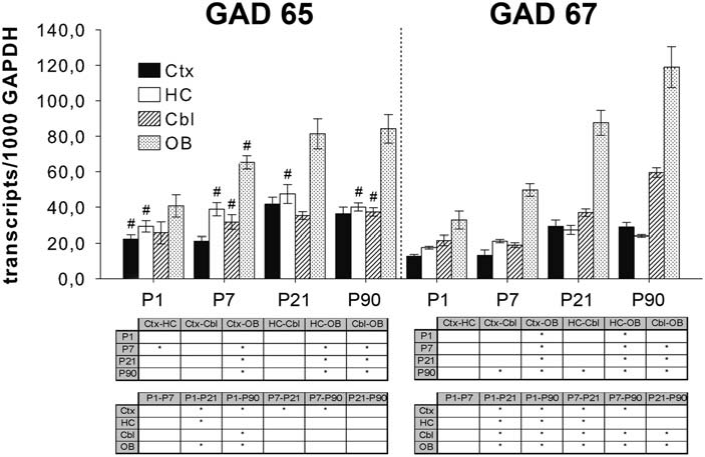
Expression of GAD65 and GAD67 in rat cortex (Ctx), hippocampus (HC), cerebellum (Cbl), and olfactory bulb (OB) at different postnatal ages. Probes were examined for GAD transcript expression by qPCR. Numbers of transcripts were calculated per 1000 transcripts of GAPDH. Data represent mean±SEM of five individual animals. ^#^ *P*≤0.05, compared with expression of GAD67 within the same region and at the same postnatal day and as evaluated by Mann-Whitney *U* test. *P≤0.05, the comparison of regions at each analyzed postnatal day (upper tables) and the comparison of ages within each region (lower tables) as analysed by one-way ANOVA followed by Tukey's post hoc test.

GAD65 messages showed a similar expression in cortex, hippocampus, and cerebellum, except of P7 when expression in the hippocampus exceeded that of the cortex. The OB significantly displayed the highest expression of GAD65 from P7 on (Fig. 1). No changes in expression were seen from P1 to P7 and from P21 to P90. Adult levels of GAD65 messages were reached in all regions between P7 and P21 (Fig. 1).

GAD67 mRNA expression was equal in cortex and hippocampus at all ages and adult levels were achieved around P21 (Fig. 1). GAD67 messages in cerebellum and OB increased continuously until adulthood. The OB presented by far the highest expression within the investigated regions (Fig. 1).

Comparing the two isoforms GAD65 mRNA was found to be the predominant form in the hippocampus from the first postnatal day throughout adulthood (Fig. 1). In cortex and OB this predominance was only observed at P1 and P7, respectively. At P90 the cerebellum showed a significantly higher expression of GAD67 compared to GAD65. In all other samples no significant differences between the two isoforms could be determined (Fig. 1).

**Figure 2:**
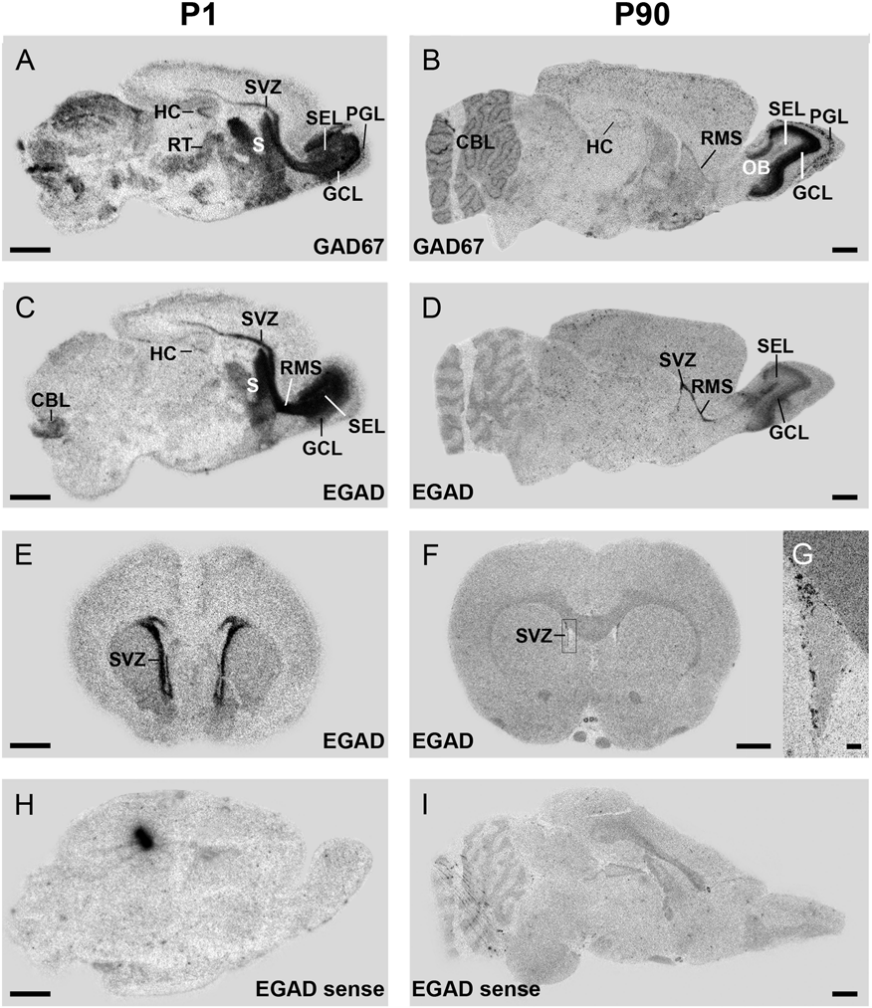
Distribution of GAD67 splice variants in rat brain at P1 (left panels) and P90 (right panels). GAD67 mRNA (A, B) and embryonic transcripts (EGAD; C–G) were detected using radioactive *in situ* hybridization. Autoradiographic film images of saggital sections showed a predominant expression of GAD67 mRNA in the olfactory bulb (OB) at both ages (A, B) and a moderate expression in the cerebellum at P90 (B). Intense labeling of embryonic messages was found in the subventricular zone (SVZ; C, E) and the olfactory bulb at P1 (C). The expression persisted until adulthood [D, F, G (enlarged SVZ from emulsion autoradiography)]. Sense probe for embryonic GAD67 mRNA was used as a negative control (H, I). Black spot in H displays an artefact. CBL – cerebellum; GCL – granule cell layer; HC – hippocampus; OB – olfactory bulb; PGL – periglomerular layer; RMS – rostral migratory stream; RT – reticular thalamic nucleus; S – striatum; SEL – subependymal layer; SVZ – subventricular zone. Scale bar A–F, I, H: 2 mm; G: 100 µm.

Radioactive *in situ* hybridization for GAD67 using a probe only detecting the adult GAD67 mRNA confirmed the data obtained by qPCR (Fig. 1), mainly the prominent expression of GAD67 in the OB and a moderate cerebellar expression (Fig. 2A, B). Additionally a remarkable expression in striatal structures and in the subventricular zone (SVZ) and rostral migratory stream (RMS) could be observed, declining from P1 to P90 (Fig. 2A, B). The OB showed a very strong expression in the granule cell layer at both postnatal times. In agreement with previous studies an intensity gradient from inner to outer layers was present [24], [25]. The periglomerular layer also showed a GAD67 transcript expression from P1 on. In contrast the strong expression in the subependymal layer at P1 could not be detected at P90 (Fig. 2B).

### Postnatal embryonic GAD67 transcript expression

**Figure 3:**
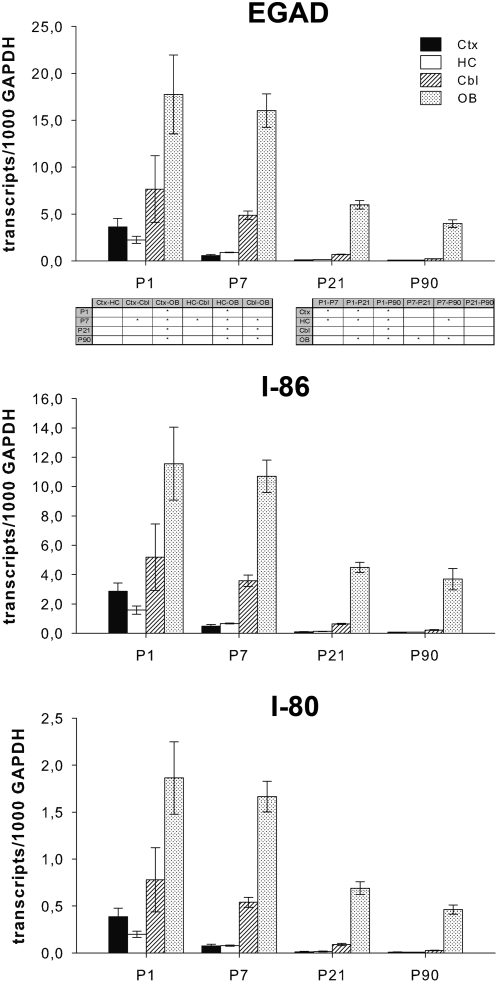
Expression of embryonic splice variants in rat cortex (Ctx), hippocampus (HC), cerebellum (Cbl), and olfactory bulb (OB) at different postnatal ages. Probes were examined for embryonic GAD67 transcript expression by qPCR. Embryonic transcripts were jointly (EGAD) and separately (I-80 and I-86) amplified. Numbers of transcripts were calculated per 1000 transcripts of GAPDH. Data represent mean±SEM of five individual animals. *P≤0.05, the comparison of regions at each analyzed postnatal day (left table) and the comparison of ages within each region (right table) as analysed by one-way ANOVA followed by Tukey's post hoc test.

Embryonic GAD67 splice variants were detectable from P1 on, but the transcript level continuously declined until adulthood (Fig. 3). Transcripts were barely detectable in cortical and hippocampal regions from P7 on whereas the reduction was decelerated in cerebellum and OB (Fig. 3). On P7 the expression of EGAD in the cerebellum was significantly higher compared to cortex and hippocampus, whereas the three regions displayed the same expression level on P21. The OB showed by far the highest transcript level at all investigated ages (Fig. 3). Discrimination of EGAD revealed about eight times more transcripts of I-86 than I-80 at all investigated ages and in all regions indicating that embryonic transcripts are dominated by I-86 (Fig. 3). The course of expression and the relative distribution of both transcripts across the evaluated regions were similar and resembled that of EGAD.

The predominant expression of EGAD in the OB demonstrated by qPCR (Fig. 3) could be confirmed by radioactive *in situ* hybridization at P1 as well as P90, with an EGAD specific probe detecting both I-80 and I-86 (Fig. 2C, D). The high expression of embryonic GAD messages in the OB at P1 diminished to a lower level at P90. While at P1 signal intensity in the subependymal layer was higher compared to the granule cell layer (Fig. 2C) expression ratio was converted throughout adulthood (Fig. 2D). Most significantly, we found a pronounced expression of embryonic GADs localized to the SVZ/RMS of P1 rats (Fig. 2C, E). In the SVZ/RMS of the adult brain their expression was weakened compared to P1 but still visible (Fig. 2D, F, G). Furthermore, moderate signal intensity could be revealed in cerebellum, striatum, and hippocampus at P1 but was vanished by P90 (Fig. 2C).

Adult and embryonic GAD67 transcripts are simultaneously expressed in the SVZ/RMS and OB. EGAD seems to be more abundant than GAD67 in the SVZ/RMS on P1 as well as on P90. In the OB of P1 rats their expression pattern is opposed; GAD67 transcript expression increases from subependymal layer to granule cell layer, EGAD expression decreases.

## Discussion

The present study provides information about the postnatal expression level and spatiotemporal expression pattern of mRNAs coding for adult and embryonic GADs in rat brain. In agreement with other studies we found GAD65 and GAD67 mRNAs to be upregulated during postnatal brain development reaching adult levels mainly within the first three postnatal weeks [26], [27]. In the adult brain GAD65 as well as GAD67 transcript expression profile was found to be critically dependent from the brain region and moreover there seems to be an ongoing regulation during ageing [26], [27]. The OB displayed by far the highest level of both adult messages compared to all other investigated regions. Furthermore our study revealed GAD65 mRNA to be the predominant isoform at all investigated ages in the hippocampus and transiently also in other regions. The adult hippocampal prevalence of GAD65 coincides with a previous semiquantitative RT-PCR study [23]. All other analyzed regions did not show an explicit prevalence of one of the adult GAD transcripts during brain maturation. In contrast to our results GAD67 transcripts have been reported to dominate in the cerebellum [27]. Notably, *in situ* hybridization studies in adult rats clearly indicate a stronger labeling for GAD67 compared to GAD65 mRNA in most of the investigated regions [1], [5], [24], [25].

In conclusion, postnatal studies of adult GAD messages by different approaches (ribonuclease protection assay, Northern blot and *in situ* hybridization, RT-PCR) in rats have led to conflicting results concerning the dominant isoform and their temporal expression [1], [5], [23]–[27] which implies a critical dependency on the region investigated as well as the method applied. In the present study we deliberately used qPCR to eliminate the variability traditionally associated with other semiquantitative methods. Despite the controversial findings concerning GAD transcript expression, GAD65 protein was found to be the most abundant isoform in the adult rat brain [28], [29].

During embryogenesis numerous studies have shown an abundance of GAD67 splice variants in the developing nervous system [12], [13], [17], [19], [22], [30], [31]. A study on mice revealed traces of EGAD at P0 which vanished in the adult brain [13]. In our experiments embryonic GAD67 splice variants were still detectable in all investigated regions during the first postnatal week continuously declining throughout postnatal development. In contrast to all other investigated regions embryonic transcripts persisted in the OB until adulthood, which could be confirmed by radioactive *in situ* hybridization and was also described previously [32]. Furthermore we found embryonic transcripts to be strongly localized to the SVZ/RMS of P1 as well as of adult rats, for the first time revealing a persistent expression in germinal niches of the adult brain.

Discrimination of EGAD revealed about eight times more transcripts for I-86 than I-80 at all postnatal ages and investigated regions, meaning that embryonic transcripts in the rat are dominated by I-86. In contrast, Szabo et al. investigated brains using semiquantitative RT-PCR analyses and showed a prevalence of I-86 at earlier developmental stages (E10.5–E11.5) whereas I-80 was found to be more abundant at later embryonic stages (E12.5–E15.5) [13]. I-80 transcripts were still detectable at P0 and were found at a very low level also in adult mice brains whereas I-86 transcripts vanished completely [13]. We suspect that the investigation of different species and a higher sensitivity of the qPCR technique used in our study are responsible for the conflicting results. Interestingly, western blotting of postnatal brain homogenates only detected GAD25 [13]. This supports our finding of a predominant I-86 transcripts expression after birth, whereof only GAD25 protein, the leader peptide including the regulatory region of full length GAD67, can be translated.

The relatively low expression of embryonic GAD67 transcripts and their rapid decline shortly after birth imply that they have only a limited relevance during postnatal development. Notable exceptions seem to be SVZ/RMS and OB where EGAD is still highly expressed as revealed in our study. Beside the dentate gyrus the SVZ is one of the regions in which neurogenesis occurs throughout life. The SVZ contains dividing neural stem cells which give rise to neuroblasts. These cells migrate along the RMS to the OB where they differentiate into interneurons (mostly presenting a GABAergic phenotype) and integrate into the synaptic network [for review, see 33]. Expression of EGAD in the adult SVZ/RMS as well as in the subependymal layer (where migrating neuroblasts arrive) and in the granule cell layer of the OB (where newborn cells are integrated) supports the hypotheses of a functional involvement in proliferation of neural stem cells, migration of neuroblasts and synaptogenesis [17], [32].

Moreover by comparing both embryonic and adult GAD67 transcripts Behar et al. noticed a sequential expression of EGAD followed by GAD67 [17]. As revealed here this still seems to be the case during postnatal development. In zones where cells proliferate and migrate EGAD is more abundant than GAD67 (SVZ/RMS) whereas in regions that primarily or exclusively contain mature neurons (granule cell layer and periglomerular layer of the OB) GAD67 expression prevails. Because embryonic GAD67 splice variants always seem to precede the adult form during development [17], [22], we suppose that expression of embryonic transcripts might be indicative of an early commitment to the GABAergic phenotype. This is indicated by the detection of GAD67 protein and GABA in neuronal progenitors of the adult SVZ/RMS [34] as well as in cell cultures from the anterior rat SVZ [35]. GABA was found to play an essential role in processes finally leading to integration of new interneurons in the OB by modulating proliferation as well as migration of neuroblasts [for review, see 36].

How exactly do embryonic splice variants influence neurogenesis, migration or even synaptic integration? Do embryonic GAD67 messages just define the GABAergic phenotype? Further research is required to answer these questions.

Investigations of activity-dependent re-expression of EGAD in the pathophysiological brain might help to elucidate their function. Szabo et al. revealed a highly selective induction of I-86 in hippocampal granule cells after kainic acid administration [23]. This transient increase in embryonic GAD67 transcription preceded the induction of adult GAD67 mRNA in hippocampal granule cells [23]. Meanwhile it is well known that after seizures or electrical stimulation, the expression of GAD67 and GABA is enhanced in the granule cell layer [29], [37] leading to a simultaneous glutamatergic and GABAergic transmission [38]–[40]. Therefore it has been speculated that I-86 transcripts might be able to regulate the expression level of the adult GAD67. Investigations of post-ischemic tissue revealed a robust expression of embryonic GAD messages in injured areas (A. Popp, unpublished data). After stroke and epilepsy adaptive processes like neurogenesis and reorganization, both accompanied by synaptic plasticity, occur [41]–[44]. Studies are underway to determine the relevance of the different GAD messages on these processes.

## Materials and Methods

### Animal procedures and sample preparation

Wistar rats of ages P1, 7, 21, and 90 (n = 8 each) were used. They were fed and housed under standard conditions with free access to food and water. All animal procedures were approved by the local government (Thüringer Landesamt, Weimar) and conformed to international guidelines to the ethical use of animals. Efforts were made to reduce the number of animals and their suffering. Rats at different postnatal ages were deeply anaesthetized with isoflurane and brains were removed after cervical dislocation.

For qPCR the cerebellum and the olfactory bulb were separated primarily. Then, coronal sections of approximately 2 mm (interaural position 4.7 to 6.7 mm corresponding to Bregma −4.3 to −2.3 mm) were dissected from the cerebrum of adult rats and the corresponding region in younger animals. Cortical specimens were taken between cuttings 3.5 and 7.2 mm lateral from the midline followed by dissection of the subjacent hippocampi. Specimens were snap frozen in liquid nitrogen and stored at −80°C. Total RNA was isolated using the RNeasy Lipid Tissue Kit (Qiagen, Hilden, Germany). RNA was quantified spectrometrically by a ND-1000 (Nanodrop, Delaware, USA).

For *in situ* hybridization brains were embedded in Tissue-Tek (Sakura, Zoeterwonde, Netherlands), frozen in isopentane at −30°C, and stored at −80°C. Coronal and saggital brain sections of 20 µm were cut on a cryostat (Leica, Bensheim, Germany), thaw-mounted onto SuperFrost Plus slides (Menzel-Glaeser, Braunschweig, Germany), and kept at −80°C until further use.

### qPCR

Cortex, hippocampus, cerebellum, and olfactory bulb of 5 animals per age were individually analyzed in this experiment. cDNA synthesis was performed using the QuantiTect Reverse Transcription Kit from Qiagen (Hilden, Germany). qPCR was carried out with the iCycler detection system (BioRad, Hercules, USA) using iQ SYBR Green Supermix (Biorad) and gene specific primers for all specific GAD transcripts and GAPDH (Glyceraldehyd 3-phosphate dehydrogenase) as the reference gene (Table 1). The following PCR protocol was applied: 50 cycles of 95°C for 30 sec, 59°C (EGAD, GAPDH), 61°C (I-86), 62°C (GAD65, GAD67) or 65°C (I-80) for 30 sec, 72°C for 30 sec. An external standard curve was used for absolute quantification. The numbers of transcripts were calculated per 1000 transcripts of GAPDH by including the criteria of the length of each specific amplicon.

**Table 1:** Sequences of gene specific primers and associated amplicon lengths and probes for *in situ* hybridization.

mRNA	Sequence 5′→3′	Product bp
	**Primers for qPCR**	
**GAPDH**	fw_TCCCTCAAGATTGTCAGCAA [45]	
	rev_AGATCCACAACGGATACATT [45]	308
**GAD65**	fw_GTACGCCATGCTCATTGCCC	
	rev_AGAGAGGATCAAAAGCCCCG	299
**GAD67**	fw_GCTGGAAGGCATGGAAGGTTTTA [30]	
	rev_ACGGGTGCAATTTCATATGTGAACATA [23]	222
**EGAD**	fw_GCTGGAAGGCATGGAAGGTTTTA [30]	
	rev_TGAGCCCCATCACCGTAGCA [30]	244
**I-80**	fw_AGTGTGGCCTCCAGAGGTTC [32]	
	rev_TGGATATGGCTCCCCCAGGAG [32]	144
**I-86**	fw_TGGCCTCCAGAGGTGATGGT [32]	
	rev_TGGATATGGCTCCCCCAGGAG [32]	146
	**Probes for ** ***in situ*** ** hybridization**	
**EGAD antisense**	ATCACCGTAGCAACCAACACTCCCTCATGTCTGATGGC [23]	
**EGAD sense**	GCCATCAGACATGAGGGAGTGTTGGTTGCTACGGTGAT	
**GAD67 antisense**	CACGGGTGCAATTTCATATGTGAACATATTGGTATTGGCAGTTGATGTC [23]	

### Statistical evaluation

All data are shown as mean±standard error of the mean (SEM) of 5 individual animals. For statistical analysis, one-way ANOVA followed by Tukey's post hoc test was used to compare the expression of each gene (1) between the various regions at each analyzed postnatal time and (2) between the different postnatal times within each region. Differences between GAD67 and GAD65 expression were evaluated by Mann-Whitney *U* test. P≤0.05 was considered statistically significant.

### Radioactive in situ hybridization

To separately detect GAD67 and embryonic GAD67 transcripts antisense oligonucleotide probes (Table 1) were synthesized and 3′ end labeled with [^35^S]-dATP (1000 Ci/mmol, GE Healthcare, Buckinhamshire, England). Corresponding sections from P1 and P90 brains were processed together. Frozen sections were fixed in 4% paraformaldehyde in 1× PBS for 15 min, acetylated in 0,1 M triethanolamine containing 0,25% acetic anhydride for 20 min, dehydrated in graded ethanol series, and air-dried. Each slide was probed with 110 µl hybridization buffer (50% formamide, 5× SSC, 500 µg/ml salmon sperm DNA, 250 µg/ml yeast tRNA, 1× Denhardt's solution, 10% dextran sulfate, and 20 mM dithiothreitol) containing 60 fmol of labeled oligonucleotide probe and incubated for 18 hours at 37°C. After hybridization, slides were washed twice in 1× SSC, 50% formamide at room temperature, four times in 2× SSC, 50% formamide at 42°C, and rinsed in 1× SSC. After dehydration, sections were exposed either to Amersham Hyperfilm MP (GE Healthcare) for 4 weeks or Amersham Hypercoat emulsion LM-1 (GE Healthcare) for 6 weeks. Coated slides were developed with Kodak D-19 developer (Sigma-Aldrich, St. Louis, USA). The specifity of the oligonucleotide probe was checked by simultaneous hybridization of sections with the corresponding sense oligonucleotide probe (Table 1). Components of the hybridization buffer and the washing procedure were used according to Szabo et al. (2000).
